# Model‐based hypervolumes for complex ecological data

**DOI:** 10.1002/ecy.2676

**Published:** 2019-04-04

**Authors:** Susan G. Jarvis, Peter A. Henrys, Aidan M. Keith, Ellie Mackay, Susan E. Ward, Simon M. Smart

**Affiliations:** ^1^ Centre for Ecology and Hydrology Lancaster Environment Centre Library Avenue, Bailrigg Lancaster LA1 4AP United Kingdom; ^2^ Lancaster Environment Centre Lancaster University Lancaster LA1 4YQ United Kingdom

**Keywords:** afforestation, Countryside Survey, Gaussian distribution, high‐dimensional, multivariate, niche

## Abstract

Developing a holistic understanding of the ecosystem impacts of global change requires methods that can quantify the interactions among multiple response variables. One approach is to generate high dimensional spaces, or hypervolumes, to answer ecological questions in a multivariate context. A range of statistical methods has been applied to construct hypervolumes but have not yet been applied in the context of ecological data sets with spatial or temporal structure, for example, where the data are nested or demonstrate temporal autocorrelation. We outline an approach to account for data structure in quantifying hypervolumes based on the multivariate normal distribution by including random effects. Using simulated data, we show that failing to account for structure in data can lead to biased estimates of hypervolume properties in certain contexts. We then illustrate the utility of these “model‐based hypervolumes” in providing new insights into a case study of afforestation effects on ecosystem properties where the data has a nested structure. We demonstrate that the model‐based generalization allows hypervolumes to be applied to a wide range of ecological data sets and questions.

## Introduction

Ecological systems are characterized by multivariate and stochastic dynamics at varying scales. Therefore it is challenging to identify when change determined by an environmental or external driver has resulted in a shift to a new state (Kowalchuk et al. 2003). Analyses that focus solely on univariate responses risk being unable to detect and predict emergent phenomena that result from the positive or negative covariance between system properties. For example, a perturbation could cause a change that is only observable in a multivariate context (Kersting 1984). Ideally, it would be desirable to consider changes in multiple ecosystem characteristics simultaneously, requiring an ability to theoretically and empirically evaluate high‐dimensional responses. Here we follow convention by referring to the high‐dimensional space of interest as the hypervolume (Blonder 2018).

A range of approaches have been developed to calculate ecological hypervolumes (see Table 1 for examples; also see Blonder (2018) for a comprehensive review), which vary in their assumptions and in their applicability to different scenarios (Blonder 2016, Junker et al. 2016). The methodologies can be split into parametric and non‐parametric approaches. Non‐parametric approaches have been widely applied and have the advantage of making no distributional assumptions, making them appropriate for data that does not correspond to a multivariate distribution. There is often a requirement for orthogonality of variables to conduct non‐parametric approaches, e.g., in the kernel density estimation (KDE) procedure conducted by Blonder et al. (2014) and the dynamic range boxes (DRB) introduced by Junker et al. (2016). Therefore researchers applying these methods often use some form of Principal Components Analysis (PCA) or allied approach prior to computing hypervolumes to ensure orthogonality (Barros et al. 2016). The drawback of this approach is that the dimensions used to build the hypervolume (e.g., PCA axes) are no longer easily interpretable in terms of the original input variables. Non‐orthogonality is not a problem for parametric approaches such as the multivariate normal model implemented in nicheROVER (Swanson et al. 2015), which incorporates information about covariance in the structure of the hypervolume. An additional advantage to parametric approaches is that they allow extrapolation and interpolation, which can be useful when sample size is low. Data set size is a particularly important problem with high dimensional problems as data becomes increasingly sparse in high dimensions (Bellman 1957).

**Table 1:** Examples of existing methods for hypervolume calculation

Method	R package	Parametric	Assumes orthogonality	Reference
Kernel density estimation (KDE)	hypervolume	no	yes	Blonder et al. (2014, 2018)
Dynamic range boxes (DRB)	dynRB	no	yes	Junker et al. (2016)
Multivariate normal model	nicheROVER	yes	no	Swanson et al. (2015)
Convex hull	geometry::convhulln	no	yes	Cornwell et al. (2006)

**Figure 1:**
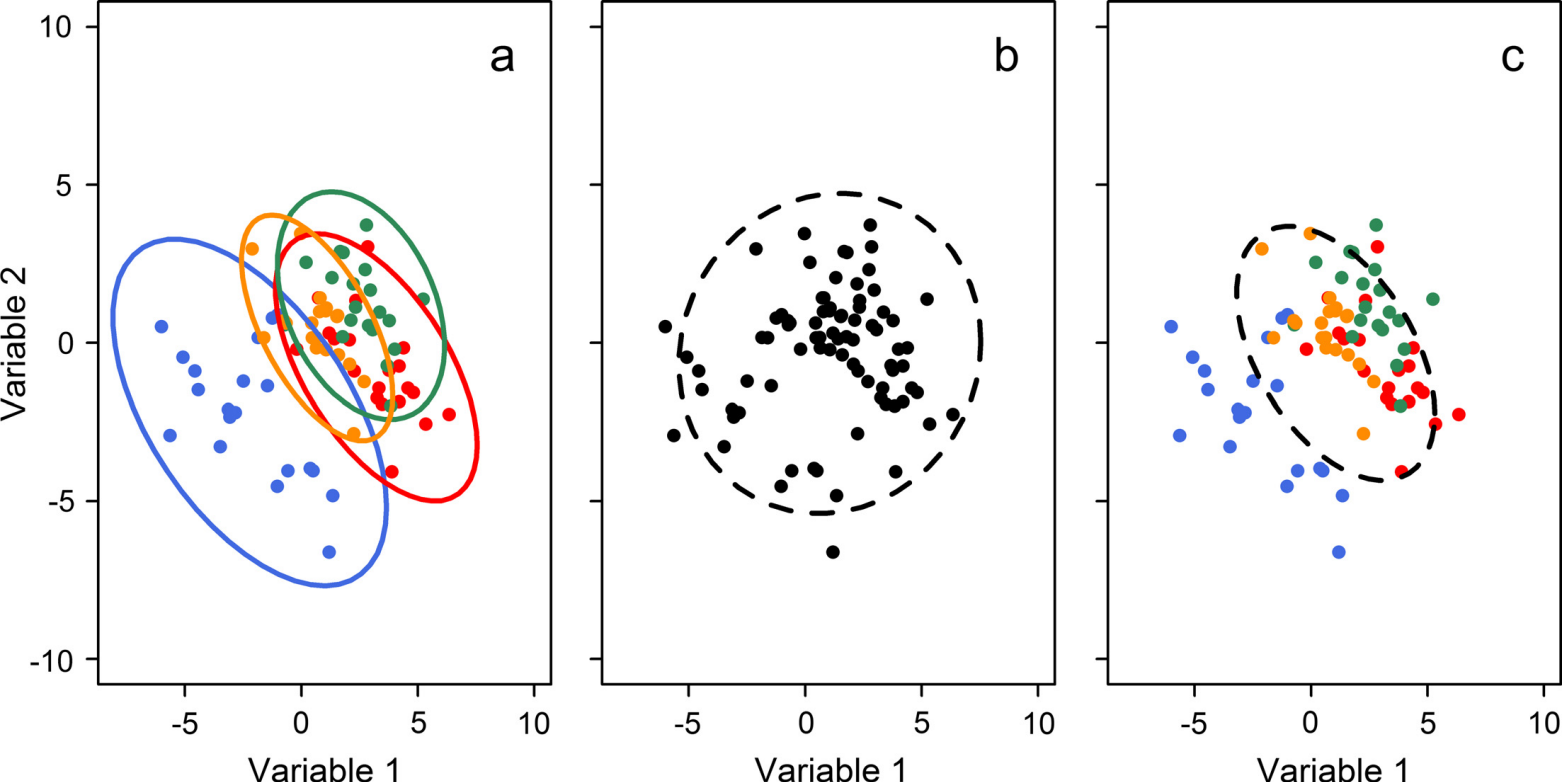
Demonstration of the concept of model‐based hypervolumes. (a) Data from different groups may share an underlying covariance structure (e.g., defined by an underlying ecological process) but have different mean values. (b) Ignoring group structure and fitting an empirical hypervolume removes any inference on covariance within groups. (c) Fitting a model‐based hypervolume can account for group differences and return the shared covariance structure.

An additional consideration is whether methods can be applied to data collected in a structured manner, for example using a nested survey method or time series. For practical reasons data collected in observational studies are often structured, e.g., multiple samples taken within catchments or regions. When data have been collected using some form of grouped design, then the hypervolume will be influenced by both the covariance within groups, and the differences between groups (Fig. 1a, b). In some cases we may want to understand the properties of the system demonstrated within groups, while accounting for differences between groups (Fig. 1c). For example, the hypervolume may reflect some underlying process shared between groups and we are therefore interested in estimating this shared covariance structure.

Accounting for data structure is possible in non‐parametric approaches by incorporating a weighting structure (Breunig 2008, Blonder et al. 2018) so that observations do not all contribute equally to the calculated hypervolume. We aim to demonstrate that multidimensional parametric approaches can be generalized to account for complex data structures in an analogous way to incorporating data structure into univariate models. If the covariance is assumed to be the same between groups, then differences in univariate means between groups should be sufficient motivation to include data structure in hypervolume construction, assuming we are interested in within‐group variation.

In what follows, we present an approach to generalize the hypervolume concept to account for data structure, which we term the model‐based hypervolume. We use simulated data to show that not accounting for structure in the data leads to biased estimates of hypervolume size, assuming that the hypervolume of interest is that obtained from the underlying within‐group covariance matrix. We then apply the method to a case study to demonstrate how model‐based hypervolumes can be calculated for nested ecological data to investigate impacts of afforestation on the ecological properties of terrestrial vegetation.

## Model Description

### Data with no structure

We initially assume that the variance–covariance matrix of a multivariate normal distribution can be used to calculate a 95% confidence ellipsoid, which is the basis of the approach to hypervolume estimation introduced by Swanson et al. (2015). We assume that the data *Y*_*i,j*_ come from a multivariate normal distribution where *Y*_*i1*_ *….Y*_*iJ*_ represents the *J* variables measured for a single ecological unit *i*. The mean of the multivariate normal distribution is described by μ_*j*_, with each variable *j* having a different mean value (Eq. (1)). The covariance matrix is given by Σ and has the dimension *J* × *J*. Matrix decomposition of this covariance matrix provides the major axes of the 95% confidence ellipsoid used to represent the hypervolume. Assuming that there is no structure in the data then Σ should approximate the covariance matrix derived directly from the data (Σ_*R*_; Swanson et al. 2015):

(1) $$Y_{i.j}∼\text{MN}\left(\mu_{j},\Sigma \right).$$

### Accounting for structured data

If observations are grouped or nested, then potential non‐independence of observations can be accounted for by adding a random effect, which varies by group. Random intercepts can be included in the above model by letting the mean of the multivariate distribution vary by each group *k* (*k* = 1,*…*,*K*) as well as by variable *j* (Eq. (2)). For each variable *j*, the means for each group *k* are drawn from a normal distribution with zero mean and variance ε (Eq. (3)). Note that overall means for each variable are not estimated, and the variance term ε captures variability in means both between groups and variables. There is no constraint therefore that means of groups within variables should be more similar than means of groups between variables. This makes it difficult to interpret ε but ensures that the within‐group variance and covariance of interest is captured in Σ, which is then used to construct the hypervolume. The covariance matrix Σ is assumed to be the same across groups and the 95% confidence ellipsoid is calculated as in the previous section:

(2) $$Y_{i,j,k}∼\text{MN}\left(\mu_{j,k},\Sigma \right)$$

(3) $$\mu_{j,k}∼N\left(0,\varepsilon \right).$$

### Probability of inclusion

The probability *q* of any new observation (Y*) being within the 95% confidence ellipsoid representing the hypervolume can be calculated from the probability distribution function of the multivariate normal distribution defined by the estimated μ_*1…J*_ and Σ. When the mean of the distribution differs by groups, μ_*j*_ can be estimated by averaging μ_*j,k*_ for each *j*. To test whether *q* is significant at any desired probability threshold, a number of simulated observations drawn from the distribution can be used to construct a cumulative probability distribution against which *q* is tested to give a probability of inclusion *p*. To capture uncertainty in the initial estimates of μ and Σ this process can be repeated for any number of μ and Σ and a summary of the inclusion probability *p* taken.

### Comparison of two or more hypervolumes

The volume of the hypervolume can be calculated using the eigenvalues of Σ to determine the major axes lengths of the 95% confidence ellipsoid. Overlap between two volumes of high dimensions is difficult to calculate precisely therefore overlap between hypervolumes is estimated by simulating a large number points from each hypervolume then testing the proportion of points from one hypervolume belonging to the other using the probability of inclusion test described above. The overlap is defined as the number of points shared divided by the total number of points simulated. This step is computationally demanding therefore the appropriate number of points to simulate should be considered based on the required precision and the available computational resources.

## Simulation Study

A simple simulation study was performed to evaluate the performance of the model‐based hypervolume in estimating the within‐group covariance structure compared to an empirical approach where group structure is ignored. The simulation study had two main components. Firstly, variable numbers of dimensions (3–7), sample sizes (10–50), numbers of groups (4–10), and between‐group variances (0–2) were assessed to check that the method was robust with a range of feasible study designs. Secondly, data were simulated with unequal variances within groups to check whether the model was robust to the assumption of shared within‐group variances. Full details of the design and implementation are given in Appendix S1.

Empirical (structure ignored) and model‐based hypervolumes were estimated for the simulated data. To produce a single metric for comparison between models the volume was computed as above, and the (estimated volume − true volume)/true volume was used as an estimate of the performance of each model. The true volume was derived from the known covariance matrix used to simulate the data.

Markov chain Monte Carlo (MCMC) estimation was used to estimate the parameters of the distribution. The prior for the covariance matrix Σ was given by a Wishart prior on the covariance matrix of the raw data Σ_*R*_ with degrees of freedom equal to the number of variables plus one. If there is no structure in the data the estimated covariance matrix Σ is expected to converge on the data defined matrix Σ_*R*_ (Swanson et al. 2015). The variance parameter ε (Eq. (3)) was not estimated but was set at 10,000, giving a weakly informative prior on group‐ and variable‐ level means. All other parameters were given uninformative priors from either normal or uniform distributions. Each simulation was repeated 500 times. All models were implemented in R version 3.4.0 and JAGS using R package rjags (Plummer 2016). An R package to run the simulations is available (see Data Availability).

## Simulation Study Results

**Figure 2:**
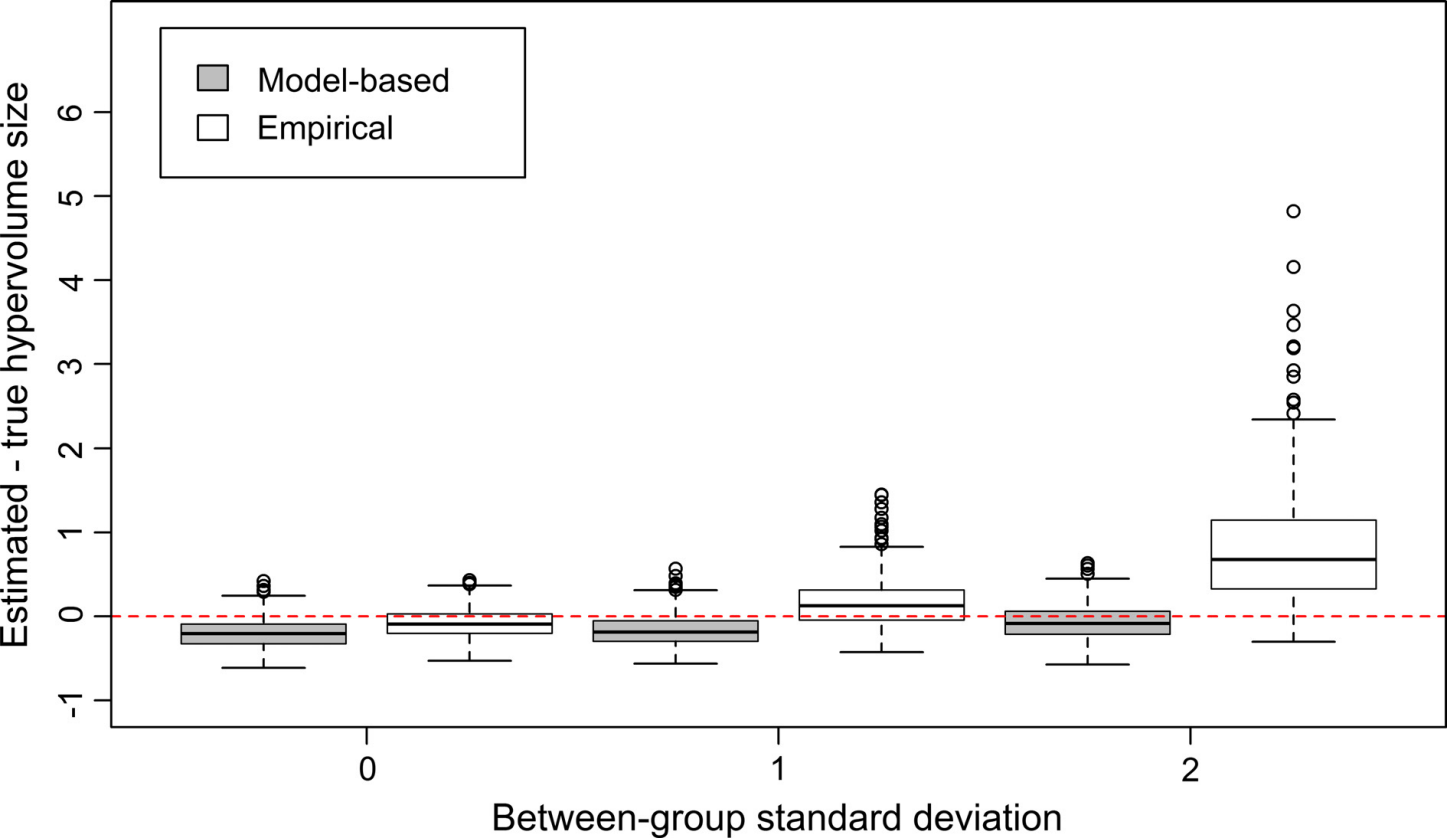
Results of a simulation study to compare true vs estimated hypervolume size with empirical and model‐based methods for nested data with varying levels of between‐group variation. Simulations shown had three dimensions, four groups and 10 observations per group. Note that the *y*‐axis is scaled by the true hypervolume size to give a relative difference. Box plot components are mid line, median; box edges, first and third quartiles; whisker, most extreme data less than 1.5 times the interquartile range from the median, and points, data more than 1.5 times the interquartile range from the median. Results for other permutations of number of observations, groups and dimensions are shown in Appendix S1.

The simulation study showed that as the between‐group variance increased, the empirical approach, where structure was ignored, increasingly overestimated hypervolume size whereas the model‐based method produced estimates closer to the truth (Fig. 2). When there was no difference between group means then both methods performed similarly and slightly underestimated the hypervolume. The potential for the empirical method to produce incorrect estimates of hypervolume size increased slightly with increasing numbers of hypervolume dimensions and numbers of groups (Appendix S1). Varying the number of observations per group did not have a large influence on the results. For the second part of the simulation study it was shown that the model‐based approach was robust to violating the assumption of equal within‐group variance (Appendix S1).

## Case Study: Afforestation Impacts on Ecosystem Services

The case study used to demonstrate the potential for model‐based hypervolumes to address interesting ecological questions comes from a long term, large‐scale study of British ecosystems known as Countryside Survey. The survey incorporates multiple co‐located observations of habitat type and vegetation composition in 2 m × 2 m plots nested within a random stratified sample of 1‐km squares across Great Britain that have been repeatedly sampled since 1978 (Norton et al. 2012). The aim of the case study was to use model‐based hypervolumes to characterize two habitats using multiple ecological metrics measured in the vegetation plots, accounting for the nested survey design. We are assuming here that we are interested in the covariance between ecosystem properties shared across 1‐km squares and therefore a model‐based, rather than empirical, approach is required.

Heath and coniferous woodland were chosen as the habitats to characterize as they were represented by a reasonable number of observations each (105 and 83 plots across 16 and 18 1‐km squares, respectively). Three variables were chosen to represent the ecosystem service indicator space in each habitat: specific leaf area (SLA), canopy height, and potential nectar production index; all were cover weighted. Data collection and processing is fully described in Appendix S2. Prior analyses of these variables have shown large variation between survey squares, highlighted in univariate plots of the data (Appendix S3), thus demonstrating the potential to apply a model‐based hypervolume. All raw data used to derive hypervolume parameters are freely available and a list of data set DOIs is provided in Appendix S4.

We quantified a model‐based hypervolume for each habitat based on the three variables described above to assess hypervolume size and overlap between habitats. Additionally, a number of plots were identified that underwent habitat transition from heathland to coniferous woodland between 1978 and 2007. We assessed the probability of inclusion of each of these plots in each habitat hypervolume and tested whether plots that had changed habitat belonged to the heath hypervolume, the coniferous woodland hypervolume, both or neither based on a probability of inclusion threshold of 0.05.

Data were centered to improve convergence, which was tested using visual inspection of the MCMC chain and via the Gelman‐Rubin diagnostic (Gelman and Rubin 1992). Models were implemented in the same manner as the simulations, using 100,000 MCMC iterations, with a burn‐in of 50,000.

## Case Study Results

**Figure 3:**
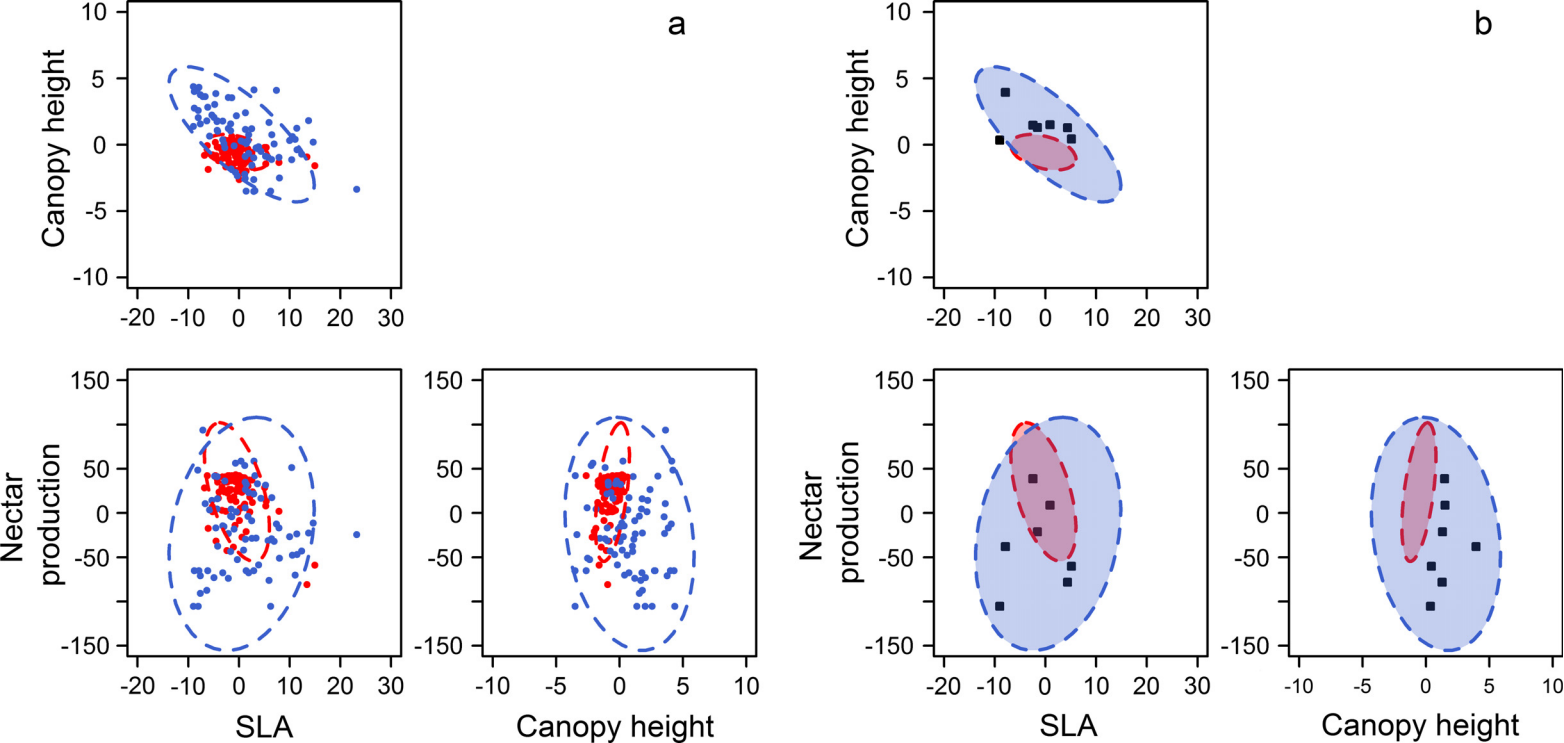
Two dimensional visualization of the heath (red) and coniferous woodland (blue) model‐based hypervolumes: panel a shows the data points used to build the hypervolumes plus the hypervolume boundaries, panel b shows the same hypervolume boundaries plus the location of the seven plots that underwent afforestation in the hypervolume space. Note that data are centered on zero but not standardized.

Two dimensional representations of the hypervolumes for both habitats are presented in Fig. 3a, showing the data points used to construct them. Seven plots were identified that had changed habitat from heath to coniferous woodland over the survey period. Fig. 3b shows variation in the position of the habitat transition plots within the hypervolumes; probabilities of inclusion and full descriptions of each plot are given in Appendix S5.

All seven habitat transition plots had a probability of inclusion in the heath hypervolume of <0.05, indicating their ecosystem properties were now more reflective of coniferous woodland. This was largely due to higher canopy height and lower nectar production in these plots than in heathland. All but one transition plot fell inside the coniferous woodland hypervolume; this plot had a combination of low SLA and average canopy height that was not typical of coniferous woodland habitat. This demonstrates the utility of the multivariate approach; the values of SLA and canopy height in this plot were not unusual for coniferous woodland when considered separately, but the combination of values placed this plot outside of the ecological range of coniferous woodland defined by the hypervolume.

When the volumes of each hypervolume were calculated, the hypervolume for coniferous woodland was found to be approximately twelve times larger than the hypervolume for heathland habitat (Appendix S6). Overlap between the hypervolumes was 34% and the coniferous woodland hypervolume almost completely contained the heath hypervolume with 99% of the heath hypervolume within the conifer hypervolume. This suggests that the range of conditions across coniferous woodlands in aboveground properties is much larger than that in heathlands, which comprise a subset of coniferous woodland conditions. The variable nature of the coniferous woodland hypervolume probably reflects the fact that areas of coniferous woodland habitat are defined by conifer cover that varies from 25% to 100%.

## Discussion

The novel methodology of model‐based hypervolumes described here allows parametric hypervolumes to be defined from structured ecological data sets when the shared covariance structure is of interest. The simulation study shows the potential for biased estimates of hypervolume properties if structure in the data is ignored in hypervolume construction.

Several considerations should be made when applying model‐based hypervolumes. First the method assumes that there is covariance shared between groups that can be estimated, reflecting shared underlying ecological processes. In addition, the method assumes the variables defining this covariance matrix are normally distributed. Deviations from normality will influence hypervolume structure, but parametric implementation of approaches for non‐normal data are challenging. Therefore a pragmatic approach is to treat data as normal where this can be reasonably assumed, and inspect deviations from this assumption in the results. In the case study, it was obvious that the nectar production index was not quite normally distributed, particularly in heathlands, leading to an area of hypervolume with no points. To demonstrate the capability of this method this was seen as acceptable, however a more in depth assessment of this data may require a different approach. Further investigation into the potential use of Gaussian copulas in hypervolume construction could be useful here (Fischer et al. 2009), otherwise a nonparametric approach might be preferred.

All multivariate approaches are subject to the curse of dimensionality (Bellman 1957). That is, increasing amounts of data are required as the number of dimensions increases. The simulation study indicated that the model‐based hypervolume performed well in up to seven dimensions, but performance in higher dimensions has not been evaluated. Hypervolumes with large (*j* > 10) numbers of dimensions are likely to require data sets of a size not achievable in most ecological investigations. In addition, although the hypervolume approach introduces tools to add insight to high dimensional data, visualization and interpretation becomes increasingly difficult as dimensionality increases. Informed and question‐led selection of variables is therefore essential.

The simulation study showed that the model‐based approach had no benefit over an empirical approach when there was no or little between‐group variance. Therefore if there is no or only slight evidence of between group differences an empirical approach may be more suitable. If there is uncertainty around this then applying both approaches and inspecting the differences in results is likely to be the most informative solution. The method also assumes that the grouping structure of the data is known. Therefore this method is most suitable for cases where nesting or grouping is defined by the study design. Future investigations could consider how uncertainty in group membership might affect interpretation of model‐based hypervolumes.

In conclusion, we present the model‐based hypervolume as a useful extension of existing methods to investigate multivariate dynamics in ecological data. The hypervolume concept considers that systems are dynamic and multidimensional, and the model‐based approach provides a flexible method for constructing the hypervolume for a wide range of ecological data sets. The simulation study shows the method is robust under a realistic set of study designs and the case study demonstrates that model‐based hypervolumes can be used to observe patterns not apparent via univariate analyses. We believe the approach could be generalizable to a broad range of ecological scenarios and could also be extended to consider temporal autocorrelation and other forms of non‐independence.

## Data Availability

Data are available from GitHub/Zenodo: https://doi.org/10.5281/zenodo.2560183
