# Supplementary material for: Model‐based hypervolumes for complex ecological data
Source: Ecology. 2019 Apr 4;100(5):e02676. doi: 10.1002/ecy.2676 (PMC6850712; doi:10.1002/ecy.2676)
Supplement: Supplementary file 1 [file ECY-100-na-s001.pdf]

Supporting Information. Jarvis, S. G., P. A. Henrys, A. M. Keith, E. Mackay, S. E. Ward, and S. M. Smart. 2019. Model-based hypervolumes for complex ecological data. *Ecology.*

Appendix S1 - Simulation study

Methods

Design of simulation study

The simulation study was designed to test whether model-based hypervolumes produced an estimate of hypervolume size closer to the truth than the empirical method ignoring group structure. In the first part, four key parameters were varied in a factorial design. These were the number of observations per group, the number of groups, the between-group variance and the number of variables/dimensions. Three levels of each parameter were combined to create a total of 81 combinations to test. The values for each level are shown in Table S1. Names given in brackets indicate the arguments used in the simulate_dataMBH function provided in https://github.com/susanjarvis501/MBH. All other arguments were left as default.

Table S1. Choice of parameter values for the first part of the simulation study

Parameter	Levels	Justification
Observations per group (nobs)	10, 20, 50	10 observations per group was seen as a minimum to obtain a sensible hypervolume, increasing numbers of observations were expected to lead to increasing precision of estimates
Number of groups (ngroups)	4, 6, 10	4 groups is taken as a minimum to define a group-level effect. Ideally, increasing numbers of groups will not affect hypervolume estimates in the model based approach
Between-group standard deviation (sdgrp)	0, 1, 2	Between-group standard deviation of 0 would indicate a situation where a model-based hypervolume was not required. Generally

		speaking, a standard deviation of 1 gives a small amount of variation between groups (some variation but smaller than within-group variation) and a standard deviation of 2 gives variation between groups approaching within-group variation. However, note that within-group variation was not fixed and could vary between 1 and 10 between simulations and variables.
Number of dimensions (ndims)	3, 5, 7	Realistically small numbers of dimensions were tested. Numbers of dimensions greater than 10 are generally not advised due to difficulties in interpretation and data requirements.

Secondly, the impact of the assumption that within-group variation was constant between groups was tested by letting within-group variances change but preserving within-group covariance. This was achieved by adding additional variation to the variances of the true covariance matrix for each group, but keeping the covariances constant. The amount of variation added was taken from a Uniform distribution with bounds of minus and plus the potential amount of additional variation, in standard deviation units. Therefore within-group variance was not set but could take a range of values up to the bounds defined. The bounds chosen for the simulation were +/- 0.5 and 1 standard deviations and were defined using the vardiff argument of the simulate_dataMBH function with variances = "variable".

The larger variance change of +/- 1 s.d. allowed within-group variances to vary substantially (e.g. if the original variance value was 4.7, within-group variances between 1.4 and 10.1 could be generated). Given that we are still trying to estimate the shared covariance matrix (i.e. before additional variation was added), this additional variation could be seen as analogous to adding a sampling effect to the data generation (i.e. the underlying variance is shared but this may not be observed perfectly). For this part of the simulation study other parameters were set at 10 observations per group, 4 groups and 3 dimensions.

Data generation

For both parts of the simulation study multivariate data were generated through the following process. The simulate_dataMBH function can be used to replicate these steps. Firstly, a random covariance is generated from which to simulate data with the following process. This ensures the corresponding covariance matrix is positive semi-definite.

1. Generate a matrix of random numbers of the required dimension (ndims) with mean 0 and standard deviation 1
2. Compute the QR decomposition of this matrix
3. Reconstruct the orthogonal Q matrix from the QR decomposition
4. Multiply the Q matrix by a vector of values of length given by ndims. Values are generated from a uniform distribution with bounds of 1 and 10 (there is an option to set this variance using the sdobs parameter but this was not used in the simulation study). Then take the cross-product of this matrix and the original Q matrix.

The resulting matrix is then treated as the true covariance matrix for resulting data generation processes. For the second part of the simulation study, the variances of the covariance matrix are allowed to differ between groups at this stage by adding random variation from a Uniform distribution with bounds as described above.

For each group in ngroups, data are generated from a multivariate normal distribution with the covariance matrix produced above. The means of the distribution are given by a random vector with length equal to ndims. The random vector is generated with a 0 mean and some standard deviation given by sdgrp. This standard deviation can be varied to represent either no differences between group means (sdgrp = 0) or increased to generate groups with increasingly variable mean values for each dimension.

The required numbers of observations per group (nobs) are then generated from the multivariate normal distribution described above using the rmvnorm function in the mvtnorm R package.

Model fitting

Models were fit in JAGS with the R code provided in the fitMBH function. Briefly, models were fitted to each simulated dataset using both empirical (grouping structure ignored) and model-based approaches as described in the main text. The volume of each modelled hypervolume was calculated and compared to the true volume calculated from the true covariance matrix described above.

In the empirical model the means of the multivariate normal distribution were given uninformative normal priors with zero mean and precision of 0.0001. The covariance matrix calculated from the data (not the true covariance matrix) was provided as an informative prior to assist convergence.

In the model-based approach, group means were given a normal prior with zero mean and variance parameter ε equal to 10,000 (provided as a precision of 0.0001 to JAGS). The estimated covariance matrix was given a Wishart prior with degrees of freedom equal to ndims + 1.

In both cases, initial values of 0.1 were given for the within-group variances and values of 0 for the covariances. Initial values were not provided for any other parameters. All model runs used 3 MCMC chains with a total of 100000 iterations and a burnin period of 20000. Chains were thinned to every 20th iteration.

Volume calculation

The mean of the posterior distribution for the covariance matrix was extracted. Because JAGS requires a precision matrix for fitting the resulting matrix was then solved to convert back to a covariance matrix. The eigenvalues of this matrix were computed and used to calculate the hypervolume size as described below:

1. Calculate the quantile of a chi squared distribution with probability 0.95 and degrees of freedom equal to ndims
2. For each dimension, multiply the quantile function by the corresponding eigenvalue and take the square root of this product. This gives the length of the semi-axes of the ellipsoid represented by the vector $a_{1 \ldots ndims}$.
3. The volume of an n-dimensional ellipsoid can then be calculated as below:

$$\frac{2}{ndims}\frac{\pi^{ndims/_2}}{\left(ndims/_2-1\right)!}\prod a$$

The estimated hypervolume size minus the true size calculated from the true covariance matrix was used as a measure of model performance. This metric was chosen as it allowed a single, easily interpretable, number to be compared across the simulation studies. Differences are presented as relative to the true hypervolume size i.e. (estimated volume – true volume)/true volume.

Results

Summarised results are presented in the main text while full results of the first part of the simulation study are detailed here in Figures S1-9. Each plot is laid out in the same way showing a side-by-side comparison of the two hypervolume construction approaches. Each dimension and number of groups is represented in a separate plot, and within the plot there are different comparisons for varying numbers of observations (nobs) and between-group variances (sdgrp). The y axis always represents the difference between the estimated and true hypervolumes.

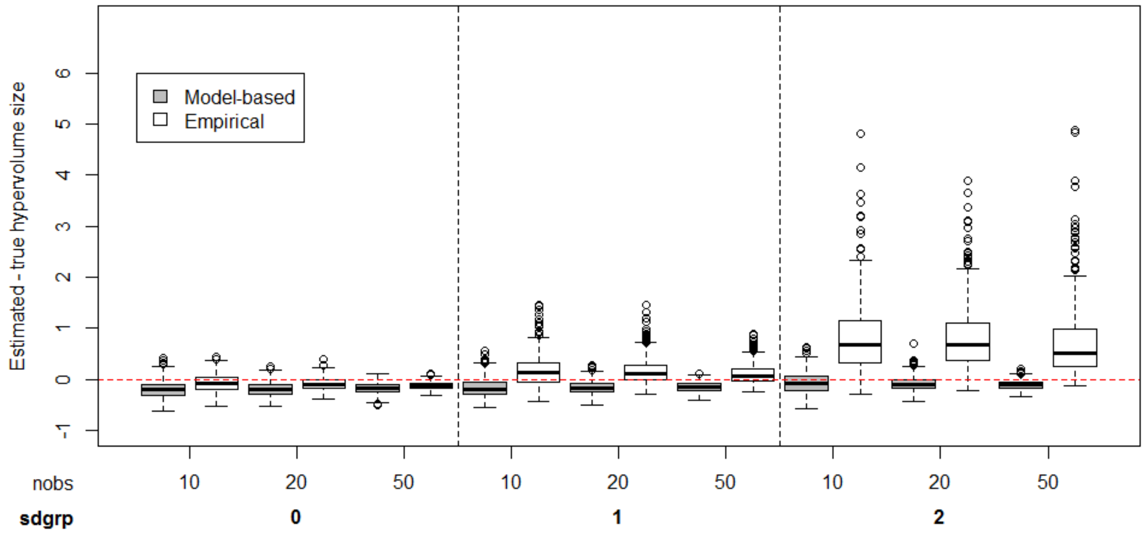

Figure S1. Simulation study results for three dimensions and four groups

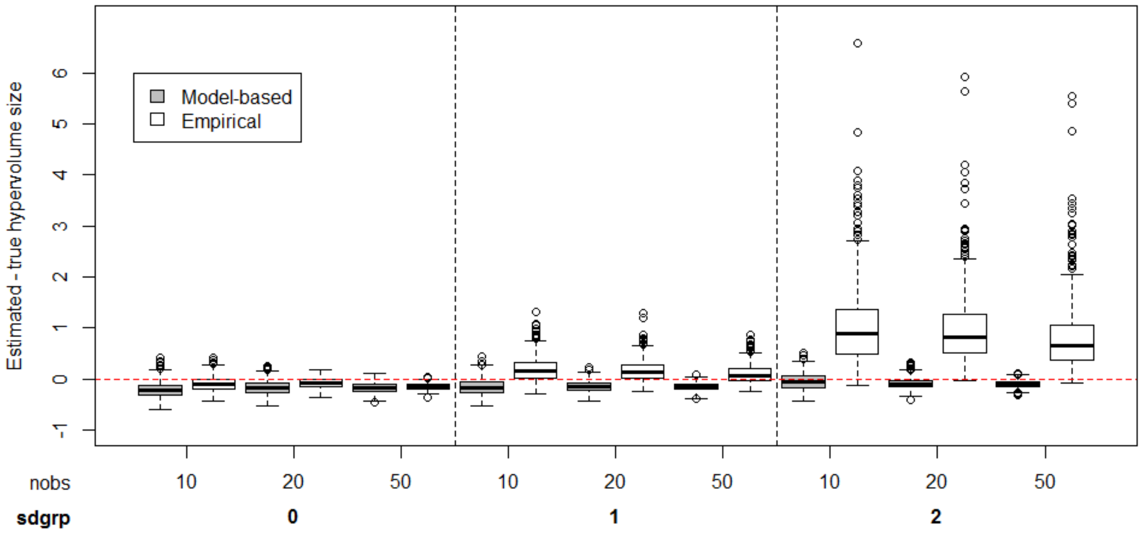

Figure S2. Simulation study results for three dimensions and six groups

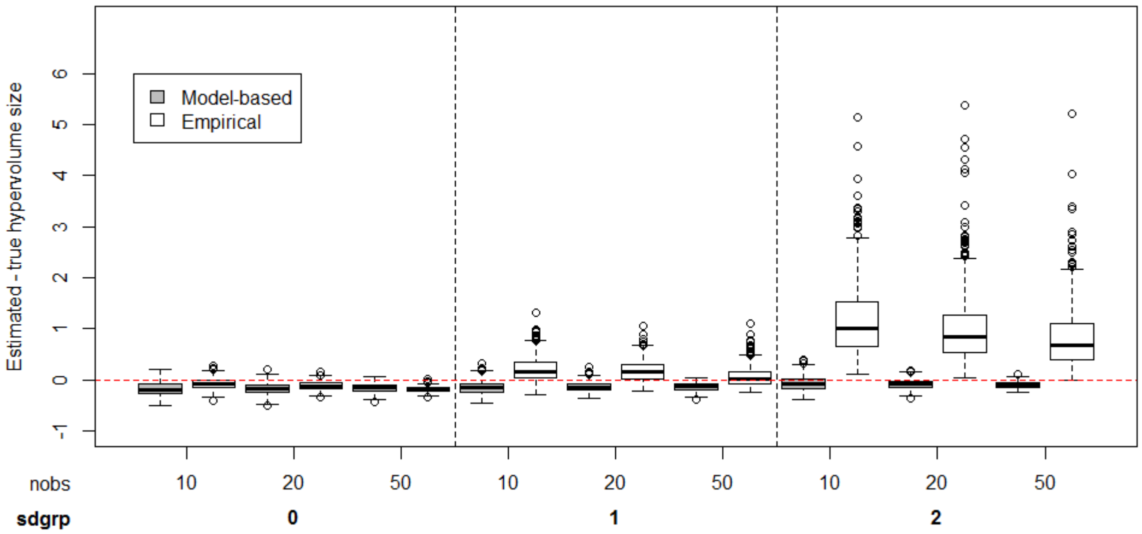

Figure S3. Simulation study results for three dimensions and ten groups

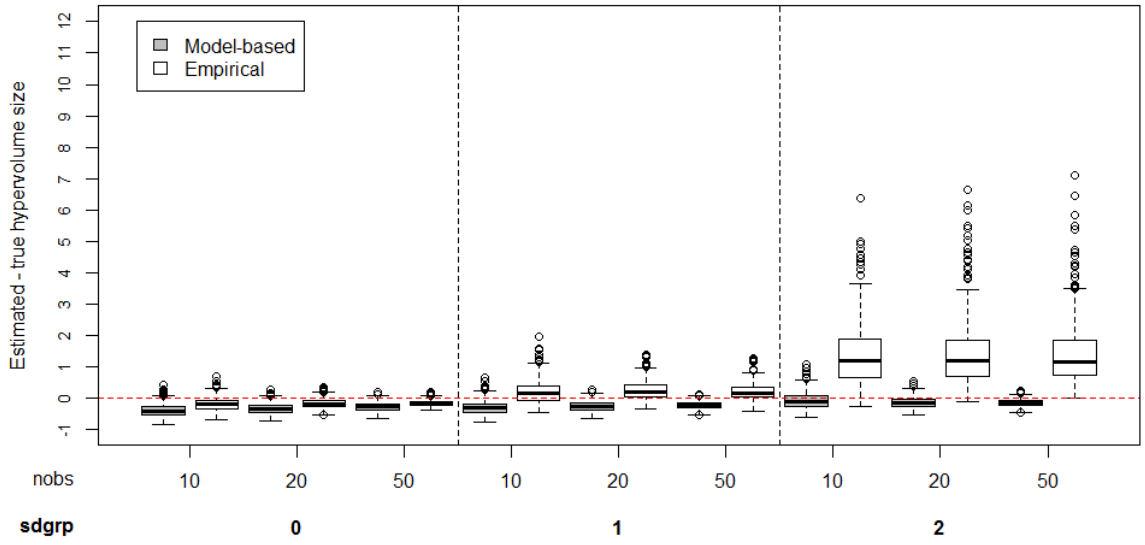

Figure S4. Simulation study results for five dimensions and four groups

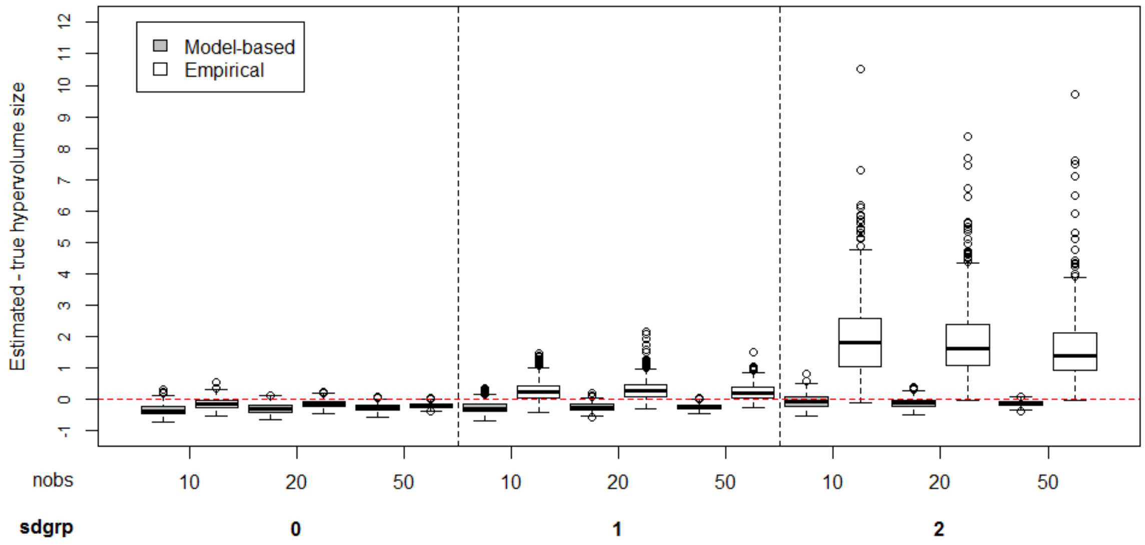

Figure S5. Simulation study results for five dimensions and six groups

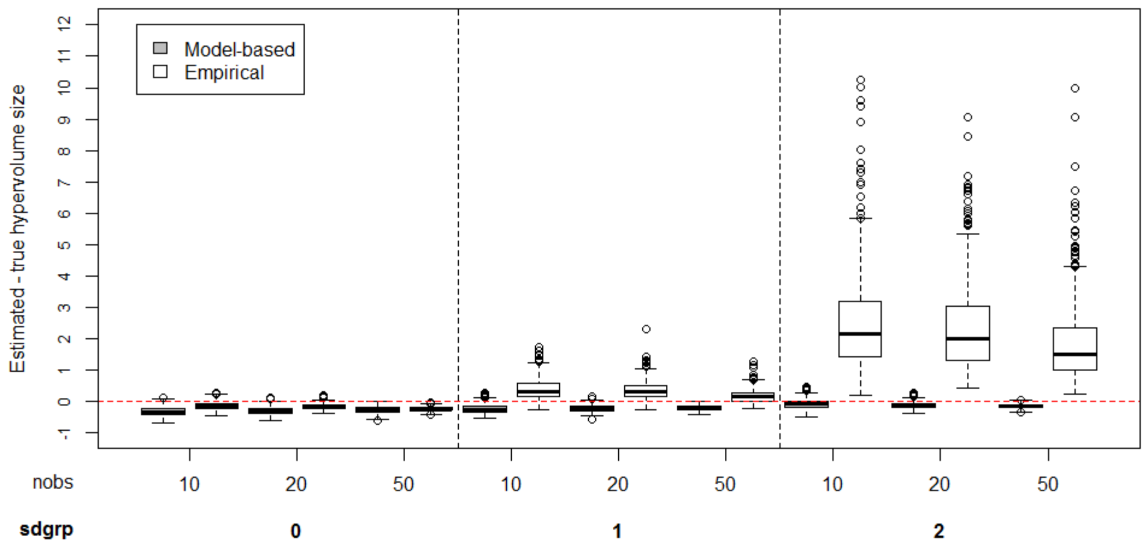

Figure S6. Simulation study results for five dimensions and ten groups

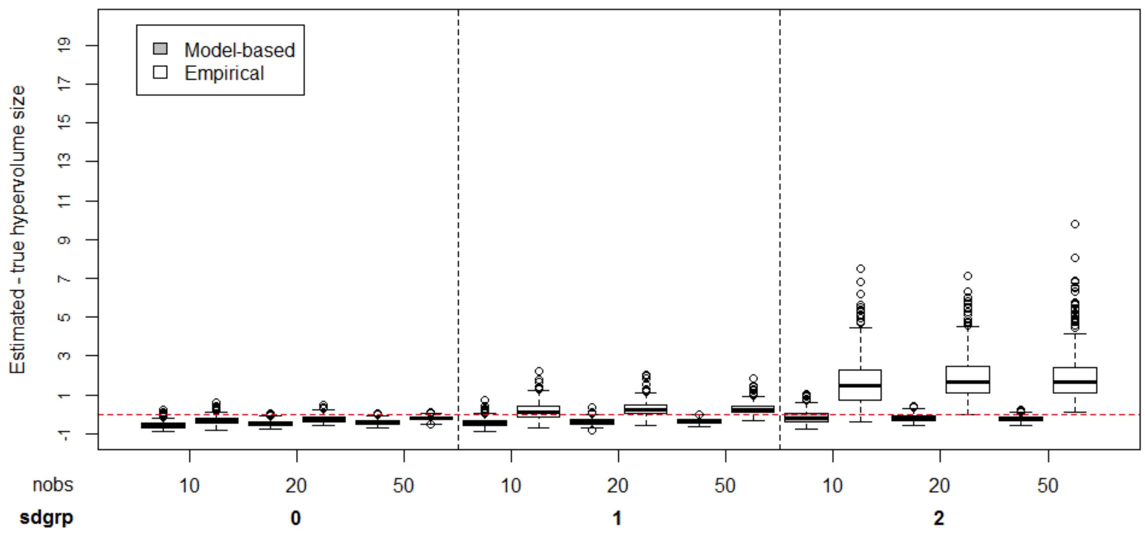

Figure S7. Simulation study results for seven dimensions and four groups

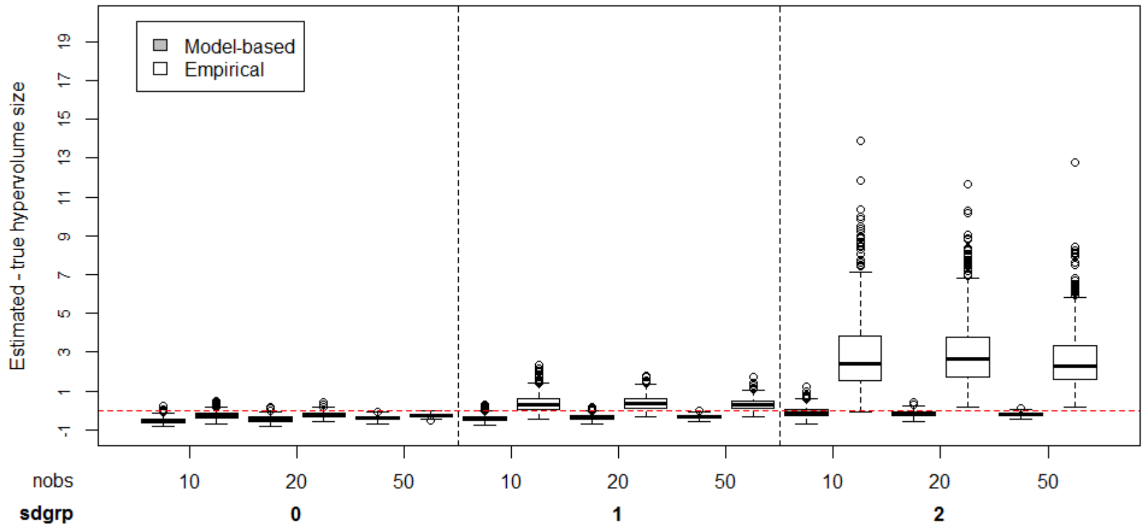

Figure S8. Simulation study results for seven dimensions and six groups

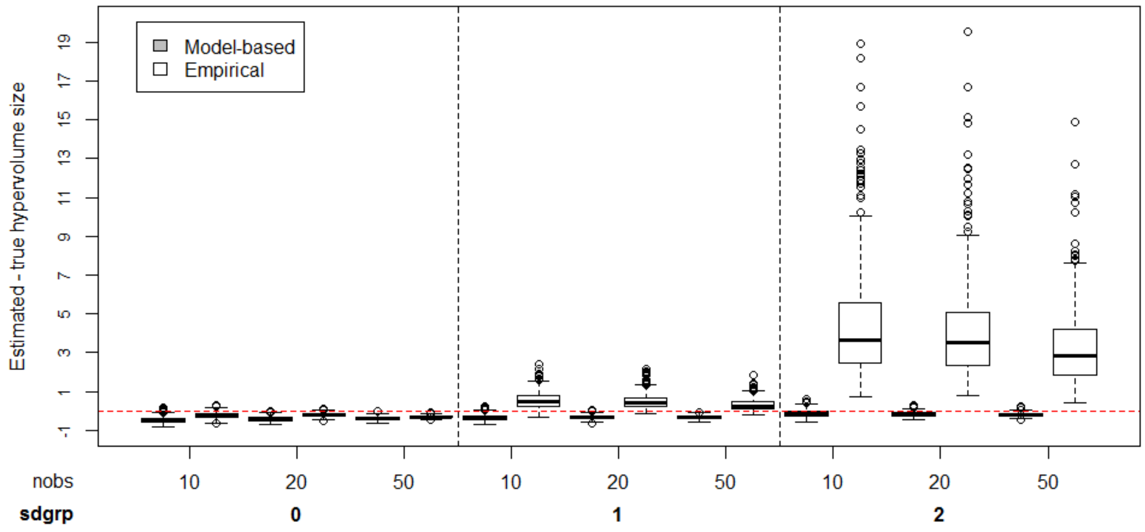

Figure S9. Simulation study results for seven dimensions and ten groups

The results of the second part of the simulation study are presented below in Figure S10. The figure is set out as above, except this time neighbouring pairs of boxplot represent different levels of difference in within-group variance while sets of six plots represent the between-

group variance (as above). In all cases simulations had 10 observations per group, 4 groups and 3 dimensions.

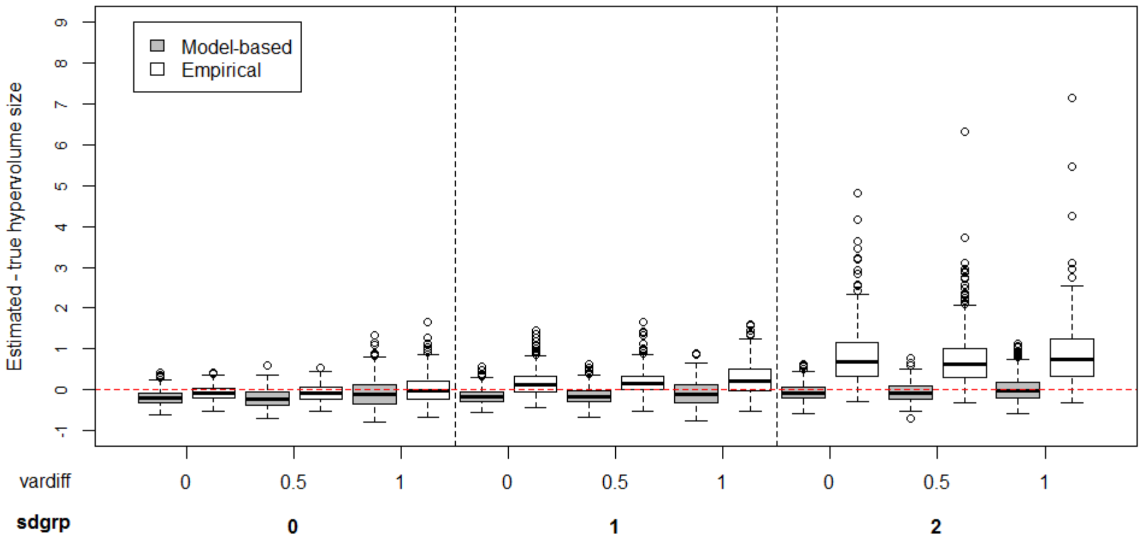

Figure S10. Simulation study results for groups with different within-group variances

Discussion

The most obvious result from the first part of the simulation study is that the empirical and model-based approaches behave similarly when there is no or little between-group difference, but the empirical approach can produce estimates far from the truth when there are differences between groups. Increasing numbers of dimensions and increasing numbers of groups lead to increasingly poor estimates from the empirical method. Changing the number of observations has relatively little effect.

The model-based method performs similarly regardless of changing observations, groups and dimensions, producing estimates close to the truth in all scenarios. Both empirical and model-based methods had a tendency to slightly underestimate hypervolume size when there were no differences between groups.

The second part of the simulation study showed that differences in within-group variance between groups had no impact on simulation outputs, suggesting both approaches are robust to inclusion of groups with different variances.
