# Supplementary material for: Model‐based hypervolumes for complex ecological data
Source: Ecology. 2019 Apr 4;100(5):e02676. doi: 10.1002/ecy.2676 (PMC6850712; doi:10.1002/ecy.2676)
Supplement: Supplementary file 2 [file ECY-100-na-s002.pdf]

Supporting Information. Jarvis, S. G., P. A. Henrys, A. M. Keith, E. Mackay, S. E. Ward, and S. M. Smart. 2019. Model-based hypervolumes for complex ecological data. *Ecology.*

Appendix S2 Case study details

The aim of the case study was to apply model-based hypervolumes to characterise heath and conifer habitats using data from the Countryside Survey. As described in the main text three variables were chosen to represent habitat properties which were derived from vegetation plots nested within 1km survey squares. The variables (specific leaf area (SLA), canopy height and nectar production) were all calculated from the vegetation cover recorded in 2m by 2m plots using auxiliary information. Further details are given by Smart *et al.* (2005) and Norton *et al.* (2012). Species mean values of each variable were combined with the cover recorded in the vegetation plots to derive cover weighted values.

Cover-weighting of each mean trait value simply allows species with greater vegetative cover in each plot to exert greater weight to the mean variable. This is consistent with the logic that the ecological effect of each trait value is amplified by the amount of biomass or plant cover in each plot relative to another plant species (Grime 1998). Dividing by the total plant cover in each plot ensures that the resulting values remain scaled to the range and units of each trait. Cover-weighted mean trait values are a routinely calculated quantity (see for example Manning et al. 2015; Smart et al. 2017).

Mean cover-weighted trait values (x_{jk}) for Specific Leaf Area (SLA), canopy height and nectar production were computed for each sampling plot j within each 1 km square k as follows;

$$x_{jk} = \sum_{i} \frac{\tau_{ijk} p_{ijk}}{\sum_i p_{ijk}}$$

where (p_{ijk}) was the percentage cover value for species i in each sample plot j within 1 km square k . Database values for SLA were extracted from LEDA (Kleyer et al. 2008) and ECPE (Grime et al. 2007). Nectar production values for each vegetation plot were calculated from the estimates

of species-specific nectar production by Baude *et al.* (2016). Plots without all three variables were not included in the analysis.

To calculate the hypervolumes, values of SLA, canopy height and nectar production were extracted for each Countryside Survey plot which satisfied two conditions: firstly that the plot was recorded as either heath or conifer in 1978 and secondly that it did not undergo any change in habitat assignment from 1978 to 2007. In addition, a number of different plot types are recorded in Countryside Survey (e.g. arable margin plots, hedgerow plots). To obtain an unbiased estimate of the characteristic properties of the two habitats only randomly located (X) plots were used in the analysis.

To serve the joint aims of increasing the number of plots with which to construct hypervolumes and to be representative of habitats across multiple years, data were pooled across three years of survey (1978, 1998 and 2007). Although there is the potential to extend the methodology to consider temporal effects, we did not do so in this case study due to the small dataset size.

Literature cited

Baude M, Kunin WE, Boatman ND, Conyers S, Davies N, Gillespie MAK, Morton RD, Smart SM, Memmott J. 2016. Historical nectar assessment reveals the fall and rise of floral resources in Britain. *Nature* 530: 85–88.

Grime, JP. 1998. Benefits of plant diversity to ecosystems: immediate, filter and founder effects. *Journal of Ecology* 86: 902-910.

Grime, J.P., Hodgson, J.G. & Hunt, R. 2007. Comparative Plant Ecology. 2nd edn. Dalbeattie, Scotland: Castlepoint Press.

Kleyer, M., Bekker, R.M., Knevel, I.C., Bakker, J.P., Thompson, K., Sonnenschein, M., Poschlod, P., van Groenandael, J.M., Klimes, L., Klimesova, J. et al. 2008. The LEDA traitbase: a database of life-history traits of the NW European flora. *Journal of Ecology* 96: 1266-1274.

Manning, P., de Vries, F.T., Tallowin, J.R.B., Smith, R., Mortimer, S.R., Pilgrim, E.S., Harrison, K.A., Wright, D.G., Quirk, H., Benson, J. 2015. Simple measures of climate, soil properties and plant traits predict national-scale grassland soil carbon stocks. *Journal of Applied Ecology* 52: 1188-1196.

Norton LR, Maskell LC, Smart SS, Dunbar MJ, Emmett BA, Carey PD, Williams P, Crowe A, Chandler K, Scott WA, *et al.* 2012. Measuring stock and change in the GB countryside for policy – Key findings and developments from the Countryside Survey 2007 field survey. *Journal of Environmental Management* 113: 117–127.

Smart SM, Bunce RGH, Marrs R, LeDuc M, Firbank LG, Maskell LC, Scott WA, Thompson K, Walker KJ. 2005. Large-scale changes in the abundance of common higher plant species across Britain between 1978, 1990 and 1998 as a consequence of human activity: Tests of hypothesised changes in trait representation. *Biological Conservation* 124: 355–371.

Smart, SM, Glanville, HC, Blanes, MC, Mercado, LM, Emmett, BA, Jones, DL, Cosby, BJ, Marrs, RH, Butler, A, Marshall, MR, Reinsch, S, Herrero-Jáuregui, C, Hodgson, JG. 2017. Leaf dry matter content (LDMC) is better at predicting aboveground net primary production than specific leaf area (SLA). *Functional Ecology* 31: 1336–1344.
