# Supplementary material for: Model‐based hypervolumes for complex ecological data
Source: Ecology. 2019 Apr 4;100(5):e02676. doi: 10.1002/ecy.2676 (PMC6850712; doi:10.1002/ecy.2676)
Supplement: Supplementary file 3 [file ECY-100-na-s003.pdf]

Supporting Information. Jarvis, S. G., P. A. Henrys, A. M. Keith, E. Mackay, S. E. Ward, and S. M. Smart. 2019. Model-based hypervolumes for complex ecological data. *Ecology.*

Appendix S3. Univariate boxplots of variables used in hypervolume construction.

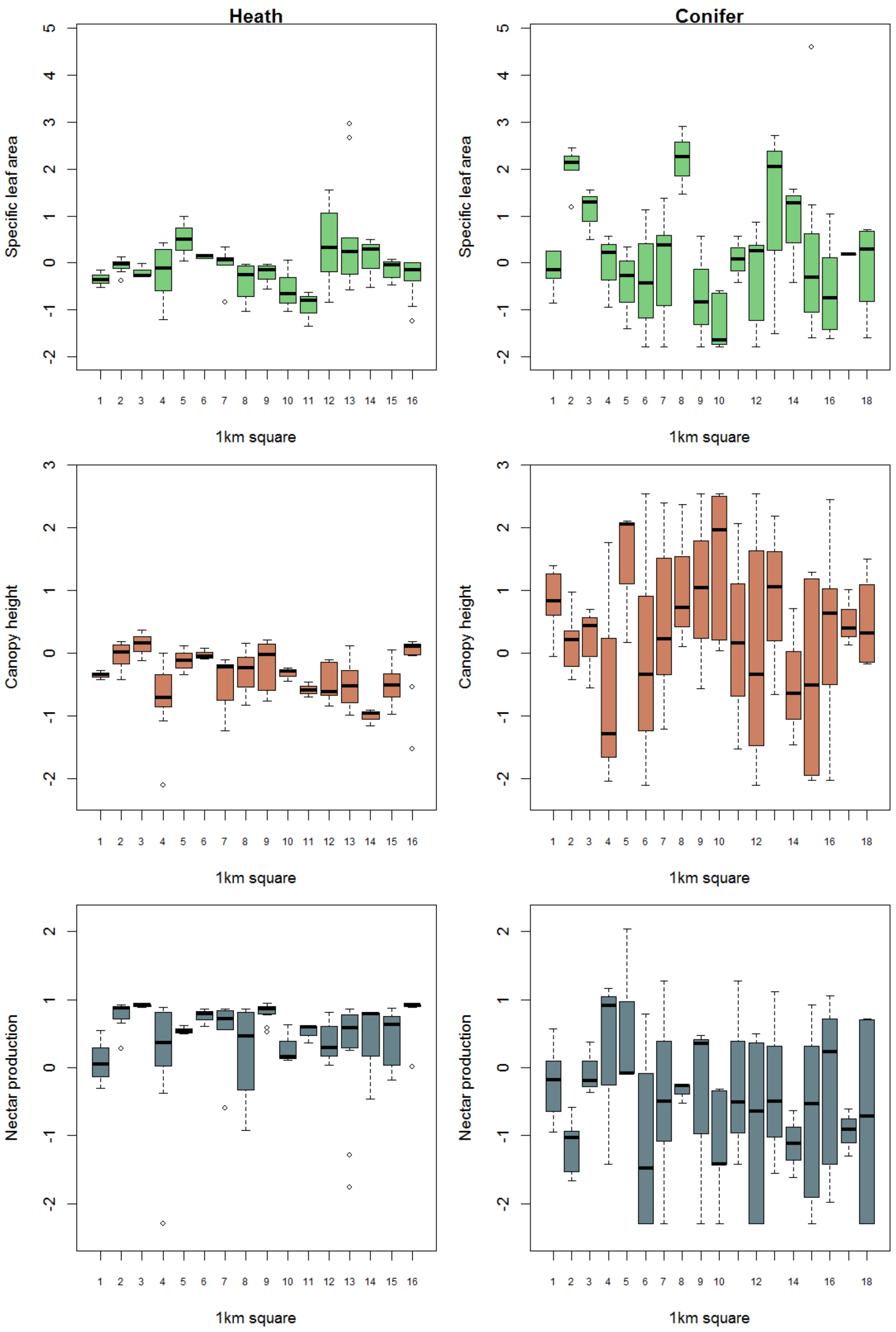

Figure S1. Univariate boxplots of the three ecosystem properties used to construct the multivariate hypervolumes discussed in the main text. It is clear that there is substantial variation due to the 1km square in which the observation is made providing justification for incorporating a random effect in hypervolume construction.
