# Supplementary material for: Model‐based hypervolumes for complex ecological data
Source: Ecology. 2019 Apr 4;100(5):e02676. doi: 10.1002/ecy.2676 (PMC6850712; doi:10.1002/ecy.2676)
Supplement: Supplementary file 4 [file ECY-100-na-s004.pdf]

Supporting Information. Jarvis, S. G., P. A. Henrys, A. M. Keith, E. Mackay, S. E. Ward, and S. M. Smart. 2019. Model-based hypervolumes for complex ecological data. *Ecology.*

Appendix S4. Data accessibility

All raw data are available from the Environmental Information Data Centre under the following DOIs.

Countryside Survey:

https://doi.org/10.5285/67bbfabb-d981-4ced-b7e7-225205de9c96 [Vegetation plot data 1978]
https://doi.org/10.5285/07896bb2-7078-468c-b56d-fb8b41d47065 [Vegetation plot data 1998]
https://doi.org/10.5285/57f97915-8ff1-473b-8c77-2564cbd747bc [Vegetation plot data 2007]

Nectar plant data:
https://doi.org/10.5285/69402002-1676-4de9-a04e-d17e827db93c [Nectar sugar values of common plant species]

Plant Att (used to derive values of SLA and canopy height) is available here:

https://www.brc.ac.uk/biblio/plantatt-attributes-british-and-irish-plants-spreadsheet

Details on the derivation of specific leaf area, canopy height and nectar production index are described in Smart *et al.* (2005) and Baude *et al.* (2016).

References:

Smart SM, Bunce RGH, Marrs R, LeDuc M, Firbank LG, Maskell LC, Scott WA, Thompson K, Walker KJ. 2005. Large-scale changes in the abundance of common higher plant species across Britain between 1978, 1990 and 1998 as a consequence of human activity: Tests of hypothesised changes in trait representation. *Biological Conservation* 124: 355–371.

Baude M, Kunin WE, Boatman ND, Conyers S, Davies N, Gillespie MAK, Morton RD, Smart SM, Memmott J. 2016. Historical nectar assessment reveals the fall and rise of floral resources in Britain. *Nature* 530: 85–88.
