# Supplementary material for: Model‐based hypervolumes for complex ecological data
Source: Ecology. 2019 Apr 4;100(5):e02676. doi: 10.1002/ecy.2676 (PMC6850712; doi:10.1002/ecy.2676)
Supplement: Supplementary file 5 [file ECY-100-na-s005.pdf]

Supporting Information. Jarvis, S. G., P. A. Henrys, A. M. Keith, E. Mackay, S. E. Ward, and S. M. Smart. 2019. Model-based hypervolumes for complex ecological data. *Ecology.*

Appendix S5. Details of habitat transition plots

Plot no	Year first recorded as conifer	Cover weighted canopy height (Grime index[1,2])	Cover weighted specific leaf area[1]	Nectar production index[1,3]	Probability of inclusion in heath hypervolume	Probability of inclusion in conifer hypervolume
1	1990	5.14	16.22	114.17	**0.0039**	0.8746
2	1998	5.10	12.85	144.14	**0.0092**	0.9576
3	1990	4.93	13.74	84.18	**0.0264**	0.8435
4	1990	4.91	19.66	27.20	**0.0018**	0.6761
5	1990	3.98	6.32	0.00	**0.0010**	**0.0053**
6	1990	7.58	7.42	67.48	**0.0010**	0.5562
7	1990	4.06	20.44	45.28	**0.0185**	0.8019

[1] Derived from dataset available with supporting metadata as Countryside Survey 2007 vegetation plot data https://doi.org/10.5285/57f97915-8ff1-473b-8c77-2564cbd747bc

[2] Height index calculated as described in Grime, J.P. et al (1988). Comparative Plant Ecology. Unwin, Hyman. London

[3] Production of index described by Baude *et al.* (2016). Historical nectar assessment reveals the fall and rise of Britain in bloom. *Nature* 530: 85-88
