# Supplementary material for: Model‐based hypervolumes for complex ecological data
Source: Ecology. 2019 Apr 4;100(5):e02676. doi: 10.1002/ecy.2676 (PMC6850712; doi:10.1002/ecy.2676)
Supplement: Supplementary file 6 [file ECY-100-na-s006.pdf]

<u>Supporting Information.</u> Jarvis, S. G., P. A. Henrys, A. M. Keith, E. Mackay, S. E. Ward, and S. M. Smart. 2019. Model-based hypervolumes for complex ecological data. *Ecology.*

Appendix S6. Volume and overlap of the hypervolumes for heath and conifer habitats.

	**Volume**	**Overlap**	**Proportion points contained in heath hypervolume**	**Proportion points contained in conifer hypervolume**
**Heath**	2929	34%	1	0.99
**Coniferous woodland**	36383		0.29	1
